# Challenges for the Development of New Non-Toxic Antifouling Solutions

**DOI:** 10.3390/ijms10114623

**Published:** 2009-10-27

**Authors:** Jean-Philippe Maréchal, Claire Hellio

**Affiliations:** 1 Observatoire du Milieu Marin Martiniquais, 3 avenue Condorcet, 97 200 Fort de France, Martinique, FWI, France; E-Mail: marechal.jean@gmail.com; 2 School of Biological Sciences, King Henry Building, Portsmouth University, Portsmouth PO1 2DY, UK

**Keywords:** coatings, marine natural products, green antifouling, environment, toxicity

## Abstract

Marine biofouling is of major economic concern to all marine industries. The shipping trade is particularly alert to the development of new antifouling (AF) strategies, especially green AF paint as international regulations regarding the environmental impact of the compounds actually incorporated into the formulations are becoming more and more strict. It is also recognised that vessels play an extensive role in invasive species propagation as ballast waters transport potentially threatening larvae. It is then crucial to develop new AF solutions combining advances in marine chemistry and topography, in addition to a knowledge of marine biofoulers, with respect to the marine environment. This review presents the recent research progress made in the field of new non-toxic AF solutions (new microtexturing of surfaces, foul-release coatings, and with a special emphasis on marine natural antifoulants) as well as the perspectives for future research directions.

## 1. Introduction

Marine biofouling can be defined as the undesirable accumulation of microorganisms, algae and animals on submerged substrates leading to subsequent biodeterioration. This is a natural process which affects both living organisms and man-made surfaces (such as medical implants, heat-exchanger tubes, pipes, cooling towers, drinking water distribution systems, probes and sensors, ship’s hulls, building materials, food-processing equipment, screens and filters, and oil industry pipelines). This review focuses specifically on marine biofouling and its control using environmentally friendly AF technologies.

Marine biofoulers are divided into three categories, depending on their impact on the increase of frictional drag (FD) of man-made immersed surfaces: a) microfoulers (bacterial, fungal and microalgal biofilms) which are responsible for 1–2% augmentation in FD, b) soft macrofoulers (macroalgae) which accounts for an FD increase of up to 10%, and c) hard macrofoulers (barnacles, mussels, tubeworms, bryozoans) leading to up to 40% increase in FD [1]. Depending on the geographical locations, the species involved fluctuate greatly accordingly to the environmental conditions (salinity, temperature, nutrient levels, flow rates and the intensity of solar radiation). Moreover, the spawning season of the organisms and consequently the pressure of fouling vary significantly according to latitude and longitude: less fouling development in winter in temperate areas (due to the reduction in day light hours and sea water temperature) with the main spawning season being from spring to late summer; marine tropical and sub-tropical areas face few variations of water temperatures and light levels, resulting in high pressure of fouling throughout the year due to a continuous period of reproduction [2].

Biofouling can lead to significant increase in the cost of maritime transportation. The globalization of production and trade are concomitant as one cannot function without the other. The scale, volume and efficiency of the international trade all have continued to increase since the 70s [3]. The importance of maritime transportation in the global freight trade is unmistakable, particularly in terms of tonnage as it handles about 90% of the global exchange [4]. The major trading routes are going via tropical and/or sub-tropical areas and consequently ships will face at some point of their voyage some very high fouling pressure. Sailing across oceans, ships are confronted with significantly different environmental conditions from tropical waters to cold or temperate waters within a few days, leading for the need of active hull protection against a wide range of organisms.

The colonisation of hulls has been linked to two major environmental pollutions which are the emissions of gas (CO_2_, CO, SO_2_ and NOx) into the atmosphere and the dissemination of potential alien species. At a given time most vessels are relatively near shore, consequently the principal amount of gas emitted is along the coastline mainly in the Northern Hemisphere, along the West and East coast of the United States, in Northern Europe and in the North Pacific [3,4]. Reduction in NOx emissions motivated by air quality concerns will tend to reduce the net warming effect due to the tropospheric ozone and CH_4_ concentrations. If these NOx reductions are greater than the corresponding increases in CO_2_ emissions, then the combined effect of NOx control could reduce the global warming impact of the international shipping [5].

Historically the most prolific vector of species translocation was hull fouling, whereby organisms attached to the hulls at one location and were carried across oceanographic boundaries during the voyage either falling off naturally in a new habitat or after the cleaning of the ship’s hulls [6]. Nowadays, ballast waters from large vessels are considered to be the dominant vector for international introductions of harmful invasive species. In addition, recreational craft are now thought to be significant secondary vectors for their spread after an initial introduction, with for example anchor and anchor chains as potential site for attachment of alien species. Invasive alien species have the ability to colonise potential habitats different from their natural habitat, invade, outcompete natives and settle permanently in new environments. They are widespread in the world and are known to affect biological diversity whether within or outside protected areas and to influence ecosystems, natural habitats and surrounding populations. All species that are non-indigenous to an ecosystem are potentially harmful, both to biodiversity and to social and economic interests [7]. The best-known and recorded examples are probably the zebra mussel introduction into the US waters and the comb jelly fish into the Black Sea [6,7].

## 2. Antifouling Coatings

AF coatings are necessary in order to avoid the colonisation of surfaces by biofoulers and consequently the high costs relative to transport delays, hull repairs, cleaning of desalination units and biocorrosion (estimated at 150 billion USD per year) [1]. During the 60s the chemical industry developed efficient AF paints using organotin compounds: tributyltin (TBT) and triphenyltin (TPT). These chemicals were highly toxic for many aquatic organisms and have been proven to contaminate the food chain and to be persistent in the environment. Since the ban of TBT-based paints (September 2008, AFS Treaty [2]), new formulation have been developed containing high levels of copper and herbicides such as Irgarol 1051, diuron, chlorothalonil, dichlorofuanid and zineb. However, even if these paints claimed to be environmentally friendly when first put on the market, there are now evidences of a widespread of these compounds in many countries (Europe, North America and Japan) with significant concentrations in marinas and harbours [8]. In addition, it has been stated that bacteria which are in contacts with AF paints can develop rapidly resistance to biocides, especially in estuaries [9,10], where most of the boats and aquaculture structures are moored. An important factor contributing to resistance is the shift of resistant bacteria to new areas due to their presence as fouling organisms in ballast waters or on ship’s hulls. It is consequently important to actively continue the development of new biocides in order to be proactive regarding these resistance issues.

The awakening of the global environmental awareness in the form of legislative measures has completely changed the way AF research is conducted nowadays. Traditionally, the industry has developed biocidal products incrementally, generating safety data as market share grows and spreading the costs over several years. However, the introduction of the Biocidal Products Directive 98/8/EC has changed all this. The regulatory authorities now require testing of new active substance before marketing authorisation [2]. The total costs have to be taken into account, for example not only preparing agreed protocols and placing studies but monitoring studies, analysis of the results, risk assessments based on exposure scenarios, dossier preparation, registration costs, task force participations, legal fees etc, as well as management activities of the directive and associated registration. For the development of new biocides, the estimated costs are as followed: toxicity studies on active substances: 1–3 M€, environmental studies & ecotoxicity: 0.6–4 M€, formulation studies: >1 M€, risk assessments/exposure scenarios expertise needed > 1m€, dossier preparation: 0.1–0.25 M€, registration fees: 0.1–0.2 M€, task forces: 0.05–0.2 M€ [11].

## 3. The Need for Development of New Non-Toxic Antifouling Formulations

There is a real need for the continuous development of new non-toxic AF formulations. An ideal AF formulation would have the following properties: permit at least five years biofouling life cycle control, durable and resistant to damage, repairable, low maintenance, easy to apply, hydraulically smooth, compatible with existing anticorrosion coating, cost effective, non-toxic to non-target species, and, effective at port and sea [12]. An interesting and promising line of research is inspired by biomimetic solutions. Indeed, most marine organisms are prone to biofouling, and colonisation of their surfaces can lead to dramatic stress. Organisms that settle on the body surface of other organisms are called the epibionts, at the opposite of the basibionts, which are the hosts. Epibiosis refers to the assemblage of epibionts on a basibiont. This complex association of species will affect the fitness of both the basibionts and the epibionts [13]. A better understanding of epibiosis and especially of its avoidance could help to design new AF solutions. Marine organisms have developed natural AF strategies which can be classified in four groups: chemical, physical, mechanical and behavioural [12]. The first three are of great interest for new AF developments and have been the basis of biotechnological research respectively on new microtexturing of surfaces, marine natural antifoulants, and foul-release coatings. They are many examples from natural fouling resistant organisms which can serve as a basis for new scientific investigations.

### 3.1. Microtexturing of Surfaces

Recently, particular attention has been paid to the physical defences of marine organisms, especially the surface topography of molluscan shells, crustose coralline algae, marine mammal and shark skin [14]. Scientists have developed methods to reproduce these microtextured surfaces (laser abrasion, photolithography, moulds & casting, and nano-particles) [14] and performed tests for their AF efficacy in the laboratory and in the field. They highlighted that fouling organisms (at the attachment phase) vary significantly in shape and size [bacteria (1 μm), diatoms (3–15 μm), algal spores (5–10 μm) and larvae of macroorganisms (120–500 μm)] [14] and that attachment points are crucial for the success of the settlement and are correlated to the size of the surface features. This complexity limits the effectiveness of surfaces to a restricted range of fouling organisms. Researchers are now developing multiple scales of topography with the goal of achieving broader deterrents effects [15]. Biomimetics models can enable an understanding of which microtextures have the best deterrence property. The new specific surfaces developed should be more efficient than the actual synthetic microtextured surfaces. The two major difficulties preventing so far the commercialisation of microtextured surfaces are the price and the impractical use for large vessels. This research area is very prolific and is progressing considerably through large consortium project such as the AMBIO project (Advances Nanostructured Surfaces for the Control of Biofouling) which aims at linking various scientific experts (chemists, engineer and biologist) with the aim of designing new wide range nano-structured coatings [16].

### 3.2. Foul-Release Materials

All marine sessile organisms use adhesive materials (with temporary or permanent capabilities) to attach to surfaces. Controlling organism’s settlement could be achieved by physically preventing adhesion [18]. Foul-release coatings have specific physical and chemical properties that affect the settlement pattern of specific biofoulers. Their efficiency varies according to the surface properties and the fracture mechanics [19]. The best anti-adhesive properties have been observed with the use of silicones as polymers [18], which on top have the advantage of being very durable. However, even if it has been shown that foul release AF treatment can inhibit the development of macrofouling organisms, they do suffer from persisting colonisation by slime [20] adhering even on vessels at speeds over 50 knots and remaining unaffected by the turbulence effect [18]. The presence of this slime increases fuel consumption and consequently CO_2_ emission. Moreover, another limitation is that foul-release coatings are efficient only when the speed of the ship produces the hydrodynamic shear needed for the loosely attached macrofouling organisms to fall off [21]. On static or slow-moving structures, the efficacy is limited to the initial stages of fouling which remain easy to remove [22].

### 3.3. Marine Natural Antifoulants

Many marine organisms, which are attached and/or soft bodied, seem not to present any physical or mechanical means of defence against possible colonisers, but do resist overgrowth by epibionts [23–25]. This ability is linked to the production of secondary metabolites involved in the chemical defence [26]. These compounds could be used as active ingredients in AF formulations.

#### 3.3.1. Discovery Process

The discovery of naturally occurring bioactive agents is based on bioassay-guided fractionation and purification procedures. The choice of the test organisms for bioassays is crucial and has to be ecologically relevant. In the previous years, most of the screening were conducted against *Ulva intestinalis* [27] and *Balanus amphitrite* [28]. But nowadays, the trend is to increase the number of organisms used in bioassays to draw a wider picture of the activity spectra of a specific compound and as well as of its mode of action [29]. Moreover, at different exposure levels, the same substance may be attractive, repellent, or even toxic, demonstrating the importance of always working with a range of concentrations [30]. The bioassays for screening new AF compounds and/or formulations can be divided into AF assays and toxicity evaluation [30]. AF assays can be performed over a wide range of organisms such as bacteria & fungi (disc diffusion assays using paper disc or glass ring methods; spectrophotometric chemotaxis assays; settlement-slide assay; biochemical assays), microalgae (inhibition of growth, settlement and adhesion), macroalgae (inhibition of the attachment of spores and zygotes and of settlement and adhesion, algal spore swimming behaviour) and invertebrates (inhibition of settlement of barnacles and bryozoans larvae, of metamorphosis of polychaete larvae, inhibition of mussels juveniles attachment and the synthesis of the phenoloxidase, behaviour of larvae) [30]. Toxicity bioassays are used to determine acute (short time exposure, typically 96 h or shorter) and chronic effects (long time exposure, from weeks to months, optimally ca. 10% of a species life-time) of a compound or mixture of compounds [31]. Toxicity is expressed as LC_50_ value, which is the concentration of a compound that kills 50% of the target organisms when administered as a single exposure. Toxicity bioassays are compulsory to get toxicological information of AF compounds. For AF formulations, toxicity tests are usually performed towards a wide range of organisms including microalgae, *Artemia* sp., oysters, barnacles, mussels, sea urchins, ascidians, fish larvae and cells of mammals. To be selected as a new promising AF compound, the new products need to have an effective concentration EC_50_ < LC_50_ [30].

#### 3.3.2. Best Sources for Bioactive Marine Natural Products

In the literature it has been stated that the best sources for AF compounds are organisms such as sponges, corals and macroalgae and/or their associated microflora and/or symbionts [25,32–34]. The active ingredients isolated and their performances against representative fouling organisms have been recently reviewed [25]. To date, purification of active products from marine organisms has yielded to around 200 molecules with variable degrees of AF activities against a wide range of marine fouling organisms [25]. Discovery of new compounds has been improved through to the continuous advances in technical innovation (increased NMR magnetic field strength, probe technology, MS bench top instruments, soft ionization and FT-MS) allowing an increase in the number of newly discovered molecules while using less quantity for structural elucidation. Moreover, the marine environment is rich in unexplored species (estimated at 1–2 million) that may have novel biosynthetic capabilities. Data analysis highlighted that AF activity is not driven by latitudinal trends, but rather by phylogenetic constraints [35]. Very promising compounds have been purified from microorganisms, macroalgae and sponges [25]. Thus, formoside and new triterpene glycosides were obtained from the sponge *Eurylus formosus* and did exhibit high and broad-spectrum activities towards bacteria, fungi, macroalgae and invertebrates [36]. Many other compounds have been purified from sponges but displayed activities against invertebrates settlement or microbial growth only. Regarding the investigation of macroalgal secondary metabolites for new AF compounds, most of the research has been focused towards Rhodophyceae and Phaeophyceae [25]. Species of the genus *Laurencia* have been extensively investigated for the production of secondary metabolites and are known to produce ca. 700 natural products, particularly bioactive halogenated compounds [25]. Regarding AF activity, the best compound obtained from this genus is the elatol which is potent against marine bacteria, and invertebrates (*Balanus amphititre* and *Bugula neritina*) at low concentration [37,38]. Concerning Phaeophyceae, the most investigated genus are *Bifurcaria* and *Sargassum* [25]. Diterpenes displaying large spectra AF activities were isolated from *Bifurcaria bifurcata* [39,40]. Interesting compounds from *Sargassum tennerimum* were shown to interfere with larval settlement of *Hydroides elegans* and biofilm formation [41]. However, active compounds are quite often produced by the associated microflora (on the surface or within the organisms), which offers a great advantage for the chemical industry as they can be grown in large volume for production of compounds. Even if no MNPs have made it yet to the AF market, an interesting product was patented a few years ago, called Biojelly® [42]. Biojelly® is a polymer that is formed on a cellulose acetate membrane immersed in seawater and harbour specific bacteria, which inhibits attachment of marine organisms such as algae and barnacles [42]. Most of the secondary metabolites are rapidly breakdown when released in the environment [43] and as a consequence their incorporation in paint a formulation is very challenging. Release rate have to be carefully controlled in order to enhance the paint lifetimes. So far, the best method develop to counter this have been perform through microencapsulation of the bioactive MNPs [43].

#### 3.3.3. Production of Bioactive Compounds

When a lead compound is discovered from the laboratory screening, field assays and paints formulation require large quantities of MNPs and the difficulties of mass production becomes a serious constraint [25]. Various options are available for a sustainable production of MNPs: chemical synthesis, controlled harvesting, aquaculture, *in vitro* production, microbial fermentation and transgenic or enzymatic production. Despite the fact that all these technologies are available, the only MNPs that have been scaled up so far are for pharmaceutical applications [24] and not yet for AF formulations.

##### 3.3.3.1. Control Harvesting

Controlled harvesting is an ideal solution when bioactive compounds are produced from unwanted biomass such as marine invasive species for examples [44,45]. Recently, it has been shown that the production of AF compounds by alien seaweeds may insure a more successful persistence in a new environment, especially when this defence appears more efficient than the ones developed by the native species [44]. Collecting organisms from the field for mass production is a cheap and convenient option but careful monitoring of the potential variation of extracts bioactivity must be carried out as it was stated that several macroalgae from temperate regions showed seasonal variation of the production of bioactive compounds [46,47], with a higher production in spring and summer. However, when bioactive compounds are produced by the native flora, harvesting in large quantity may cause a negative ecological impact, and, in that case other solutions are preferably chosen.

##### 3.3.3.2. Chemical Synthesis

The production of chemicals, fuels, pharmaceuticals, flavours and fragrances is routinely performed with catalytic tools such as enzymes, inorganic and organic catalysts [48]. However, present state-of-the-art processes for synthesis of natural products are considered highly inefficient [49]. The exact build-up of functional groups within a complex molecule still represents a challenge [48]. Inspiration from biosynthetic pathways and the natural reactivity of functional groups have been used constructively in new approaches to chemical synthesis of MNPs without protecting groups [50]. When chemical synthesis is successful, the next step is the generation of synthetic chemical analogues of the originally isolated molecules, which gives rise to a complete family of active AF compounds and, often, candidates with a more suitable bioactive profile. The synthetic analogues provide valuable information on the structure versus activity relationship (SAR), which is used to additionally refine the chemical structure and to improve the AF properties (maximum activity and minimum side effects). It is done by adding or deleting chemical groups in order to identify and determine which of the chemical groups are responsible for the biological activity. The SAR should be a consensus between the level of activity and the cost of synthesis. In order to keep the cost of synthesis as low as possible, the lead compound production must be performed by avoiding toxic, expensive and unstable reagents, as well as avoiding the use of processes patented by competitors. Another issue is when hazardous wastes are produced as a result of the synthesis, what can have significant environmental and economic impacts. Synthetic molecules are increasingly produced by combinatorial chemistry approaches, in which a common core is elaborated by attaching combinations of fragments to reactive sites on the core’s periphery [51]. In the construction of a synthetic combinatorial library, various elements (R1, R2 and R3) are attached to a common skeleton. If ten versions of each diversity element are used, the library contains 1,000 different molecules, each with a different combination of R1, R2 and R3. Several natural-product-like combinatorial libraries have been synthesized [52]. Combinatorial biosynthesis uses the manipulation of biosynthetic machinery to accomplish much the same goal, but with greater control over core elements. However, diversity-oriented synthesis, which combines the strengths of combinatorial multiplexing and core variability, is emerging as a powerful technique for finding biologically active small molecules [53]. Natural products can be classified according to shared scaffolding elements, which reflect the strategies for their assembly by pathways of biosynthetic enzymes in the producer organisms. The building blocks for natural products are most often the monomer constituents of primary metabolic pathways, which are shunted into the secondary pathways when a particular metabolic channel is opened. When monomers dedicated to secondary metabolic pathways are required, they are produced by a ‘just-in-time’ cellular-inventory strategy [54]. Elucidation of the gene and enzyme involved may lead to transgenic and/or enzymatic production of the bioactive natural products.

##### 3.3.3.4. Aquaculture and Mariculture

Aquaculture and mariculture can be used as a sustainable means for production of MNPs [55]. Due to an increase in the research efforts, aquaculture of marine invertebrates for production of MNPs is now a reality with cultivation, for examples, of ascidians, bryozoan, sponges and gorgonian corals [55]. However, economic projections suggest that in-sea culture is a cost-effective option for the supply of MNPs and is a good option as an intermediate measure until chemical synthesis or fermentation technology is developed. From a mass production point of view, algae and microorganisms seem so far to be the best candidate for providing a sustainable source of AF compounds because they can be either cultivated or harvested (*e.g.*, for algal invasive species and can be used for local production of AF compounds). For algae, it has been demonstrated that in temperate region, the seasonality pattern of production of bioactive compounds has to be considered and monitored [46]. In order to reduce this complexity, aquaculture in natural environments or outside ponds should be avoided, and controlled conditions should be preferred for temperate species. For tropical species, which are less susceptible to seasonal change, aquaculture could be done in open ponds and in the open-sea and so will be less expensive and would represent a better investment. Concerning the mass production of marine microorganisms, there is currently a global political drive to promote white (industrial) biotechnology as a central feature of the sustainable economic future of modern industrialized societies [56]. Because of special growth requirements, only a minority of the marine microorganisms could be cultured so far. Less than 5% of the viable bacterial cells in marine sample ultimately grow under standard culture conditions. Nowadays, most productions are still carried out at the shake-flask level with investigations in bioreactor engineering and fermentation protocol design in progress. New culture methods should take into account the environmental parameters associated with the habitat sampled. Improvement of the isolation method and fermentation are necessary.

Microalgae can be grown in open-culture systems, such as ponds, lakes, raceways, or in highly controlled closed-culture systems. Certain microalgae are very suitable for open system culture where the environmental conditions are very specific (high salt or high alkaline ponds). The extreme nature of this environment severely limits the growth of competitive species that may contaminate the culture. The advantage of such systems is that they are generally a low investment, very cost-effective and easy to manage. Open-culture systems take advantage of natural sunlight and are totally subject to the vagaries of weather unless some form of shading system is utilized. At the opposite, closed-culture systems require significantly higher investments and operating costs. They are independent of all variations in agro-climatic conditions and are very closely controlled for optimal performance and quality.

## 4. Perspectives for New Research

### 4.1. Broad Range Activity vs Specificity

TBT-containing AF paints have dominated the world market during 40 years. Since its restriction and ban [2], it is estimated that around 18 alternative broad-active compounds are used worldwide as biocidal AF additives [57]. However, due to increasing toxicity concerns, some of these biocides have been restricted in a number of EU countries (especially for small vessels) [57] and more countries worldwide will follow these restrictions. Broad-spectrum AF coatings are necessary for the international maritime traffic, but environmental consideration may orientate the research effort on developing “regional” paints formulation with targeted activities towards species from particular local environment or geographical location. These formulations could be marketed for the leisure sailing and motor yachts, as well as for any permanent structures (such as pontoon, buoys and aquaculture equipments). A limitation to this perspective is that most of the current bioassays have been developed towards tropical species and only a few do target temperate or cold-water species [30]. However, recent data showed pronounced differences in MNPs effects even in closely related species: for example a compound active against *B. amphitrite* may be not active against *S. balanoides*, thus demonstrating that it is not possible to extrapolate bioactivity results (Hellio and Maréchal, unpublished data). This argues for the development of new AF bioassays targeting cold and/or temperate species. In order to reach a good level of AF activity at non-toxic concentrations against non-target species, the only option might be a combination of different MNPs and technologies. The synergistic effect of this mixture could represent the next breakdown for “environmentally friendly” AF paints formulations. Such approach is used in medical research where it has been stated that the combined action of two drugs may be more powerful than their simple effects added together [58]. So far, few AF studies have followed these approaches. The ideal solutions may be a combination of microtextured-surface associated to foul-release materials along with MNPs [59].

### 4.2. New Environmental Challenges and Paints Formulation Evolution

Human are always conquering new areas for developing industry. Recently, the oil and gas industries have been blooming into the offshore environment and deep waters, creating new niche for formation of biofouling communities [60]. Marine biofouling can have a number of detrimental effects on offshore installations such as the obscuration of structures (resulting in extended inspection time), creation of microenvironments that may promote corrosion, physical obstruction, additions to weight loading, and increased hydrodynamic loading. Each of these factors has large cost implications in relation to the inspection, maintenance and repair of offshore installations [61]. Biofouling is governed mainly by salinity, temperature, nutrient levels, flow rates and light levels. All these factors vary seasonally, spatially and with water depth [60]. In the sub-tropical and tropical areas, man-made structures located within the subtidal zone (low-tide mark to 200 m depth) and in the upper regions of the bathyal zone (200–2,000 m depth) can be subject to the formation of considerable macrofouling communities within weeks to months [61]. The move towards deep-water oil and gas production (abyssal zone 2,000–6,000 m) has led to the development of research program to study the colonisation of such immersed structures [60–62]. It was demonstrated that increases in water depth resulted in changes in the physical and chemical environment less favourable to the development of fouling communities (concomitant to the depletion in nutrient concentrations, light levels and water temperature, and, the augmentation of pressure). However, photosynthetic fouling organisms are replaced by suspension and filter-feeding organisms including barnacles, sea anemones, sponges, tube-worms and bryozoans [60]. The lack of hard substrates in deepwater environments makes man-made structures such as sub-sea production systems extremely attractive to colonising organisms [63]. As fouling does occur in deep water, but at a low pace, it can be a significant problem on long-term immersed structures. A better knowledge of the biodiversity of these communities and settlement pattern will help to design specific AF protection.

Another challenge that the AF industry may face is linked to the potential environmental changes caused by global warming [64]. The mains effects of global warming on marine environments are expected to be an increase of temperature and input of freshwater which will subsequently disrupt the equilibrium [64]. The predicted impact of global warming on biofouling communities have been reviewed recently [64] and the main conclusions are that species that make use of calcium could be replaced by non-calcareous species (tunicate and algae), with a dominance of UV resistant species that can tolerate low salinity [64]. In order to be proactive regarding the possible change of communities, AF coatings should be now systematically screened against more soft-bodied organisms such as tunicates, sponges and algae. This will necessitate the development of new bioassays. Moreover, the new environmental conditions could have a negative impact on the actual paint performance such as increased polishing and biocide leaching rates, earlier paint exhaustion, potential changes on the hydrolysis rate of specific binders [64,65]. Development of AF coatings, which perform well at altered climate conditions, should be an important direction of research.

## 5. Conclusions

Even if the research on new improved AF solutions is very active, so far the perfect solution has not been found. The most promising areas of development are the production of bioactive substances from marine organisms, which could be formulated within paints matrix and the creation of new surfaces that cannot foul [66]. The best solutions would certainly be a mixture of these two technologies [17]. However, in order to develop better solutions, we need to gain more understanding on the organisms’ adhesion strategies as well as on the interspecies relationships in benthic communities.
